# The Pharmacokinetics of The Biliary Excretion of Ciprofloxacin

**DOI:** 10.1155/1989/41415

**Published:** 1989

**Authors:** C. S. Ball, J. Mck. Manson, Fiona Reid, D. E. F. Tweedle

**Affiliations:** 1 Department of Surgery Withington Hospital Manchester M20 8LR UK; 2 Department of Medical Statistics Withington Hospital Manchester M20 8LR UK

## Abstract

The pharmacokinetics of ciprofloxacin excretion have been studied in 54 patients undergoing biliary and pancreatic operations with and without obstruction of the common bile duct. High concentrations were achieved in common duct bile within 20 minutes of intravenous injection and persisted for over 3 hours after 100 mg and for over 8 hours after 200 mg. The concentration of ciprofloxacin in the bile of functioning gall bladders was much greater than that in the common duct bile. Remarkably, it was identified in therapeutic concentrations in the bile of obstructed ducts. This and the rapid fall from initially high venous concentrations probably reflect diffusion from the circulation as a result of the exceptional tissue penetration. A unique feature of this study was the finding of clinically significant concentrations in the bile of obstructed ducts.

Two patients developed wound infection and no side effects were observed. The broad spectrum antibiotic ciprofloxacin has potential as a useful agent for prophylaxis in biliary surgery maintaining biliary and venous concentrations in excess of the MIC_90_ for most biliary pathogens for more than 8 hours.


*HPB Surgery* 1989, Vol. 1, pp. 319–327
Reprints available directly from the publisher
Photocopying permitted by license only
© 1989 Harwood Academic Publishers GmbH
Printed in Great Britain

### THE PHARMACOKINETICS OF THE BILIARY EXCRETION OF CIPROFLOXACIN

C.S. BALL, J. McK. MANSON, ‡FIONA REID and *D.E.F. TWEEDLE

*Departments of Surgery and ‡Medical Statistics, Withington Hospital, Manchester M20 8LR*

*(Received 12 October 1988)*

The pharmacokinetics of ciprofloxacin excretion have been studied in 54 patients undergoing biliary and pancreatic operations with and without obstruction of the common bile duct. High concentrations were achieved in common duct bile within 20 minutes of intravenous injection and persisted for over 3 hours after 100 mg and for over 8 hours after 200 mg. The concentration of ciprofloxacin in the bile of functioning gall bladders was much greater than that in the common duct bile. Remarkably, it was identified in therapeutic concentrations in the bile of obstructed ducts. This and the rapid fall from initially high venous concentrations probably reflect diffusion from the circulation as a result of the exceptional tissue penetration. A unique feature of this study was the finding of clinically significant concentrations in the bile of obstructed ducts.

Two patients developed wound infection and no side effects were observed. The broad spectrum antibiotic ciprofloxacin has potential as a useful agent for prophylaxis in biliary surgery maintaining biliary and venous concentrations in excess of the $MIC_{90}$ for most biliary pathogens for more than 8 hours.

KEY WORDS: Pharmacokinetics, bile, ciprofloxacin.

### INTRODUCTION

Septic complications are common after biliary surgery performed without prophylactic antibiotics[1–3] but are uncommon when prophylactic antibiotics have been used[2,4]. Ciprofloxacin is a quinolone related to nalidixic acid but having a broader spectrum of antimicrobial activity and fewer side effects[5]. Its spectrum of activity suggested to us that it might be useful as a prophylactic antibiotic in patients undergoing biliary surgery.

### MATERIALS AND METHODS

Sixteen male and 38 female patients aged 22–86 years gave written informed consent for entry into the study. Seventeen showed some degree of obstruction to the drainage of bile due to stones (9), pancreatic carcinoma (5), carcinoma of the ampulla of Vater (1), carcinoma of the common bile duct (1) and acute pancreatitis (1).

Ciprofloxacin was injected intravenously as a single dose at varying times up to 8 hours before anaesthetic induction. Thirty-two patients were given 100 mg and 22 were given 200 mg. At operation samples were taken of common duct and gall

Correspondence to: D.E.F. Tweedle, Department of Surgery, Withington Hospital, Manchester M20 8LR, UK

bladder bile and venous blood. The time of infusion and sampling was recorded and samples were stored in oxalate at −20°C until analysed by high performance liquid chromatography.

### RESULTS

Two patients suffered wound infections and no adverse reactions to ciprofloxacin were observed. One patient developed an infection 9 days after cholecystectomy and *Staphylococcus aureus* of a phage type known to be prevalent in the hospital was isolated suggesting that this infection was exogenous. Another occurred 14 days after cholecystectomy, 1 week after discharge from hospital and swabs for culture were not obtained.

Patients with an obstructed common bile duct (CBD) were classed into partial or complete obstruction (Table 1) on the basis of the clinical picture, operative findings, radiological investigations and degree of disturbance of liver biochemistry. In 5 patients the common bile duct sample was insufficient for assay.

A series of mathematical models were examined to determine whether the time since infusion and/or degree of common bile duct obstruction could be used to predict ciprofloxacin concentrations in both venous blood and the common bile duct. The models were developed using the method of maximum likelihood[6]; significance of the model parameters was set at the conventional 5% level. Optimum prediction was obtained when both the ciprofloxacin concentrations and times since infusion were converted to natural logarithms. The calculations were performed using the GLIM 3.77 computer package[7] at the University of Manchester Regional Computer Centre.

### *Venous Blood*

The prediction model for venous blood concentrations is detailed in Table 2 (i and ii). The agreement between the model predictions and the observed assay values for the 100 mg dose are shown graphically in Figure 1. A similar representation of the 200 mg dose data is shown in Figure 2; the numbers of patients with partial and total obstruction receiving 200 mg were too small for the derived prediction curves to be considered acceptable for clinical use, so these curves were not plotted.

Venous blood concentration was found to fall exponentially with time. Concentrations were consistently higher with the 200 mg dose than with the 100 mg dose. This difference was significantly more marked in the presence of partial obstruction ($p = 0.002$).

**Table 1** Classification of degree of common bile duct obstruction.

| Dose | 100 mg | | | 200 mg | | |
|---|---|---|---|---|---|---|
| Obstruction | Nil | Partial | Complete | Nil | Partial | Complete |
| No. of patients | 19 | 8 | 5 | 18 | 2 | 2 |

**Table 2** Analysis of venous blood concentrations – derivation of predicted values

(i) *Analysis of variance table*

| *Source* | Degrees of Freedom | Mean Square | F-ratio | Probability |
|---|---|---|---|---|
| Time | 1 | 21.160 | 99.864 | 0.001 |
| Dose | 1 | 5.489 | 25.905 | 0.001 |
| Obstruction | 2 | 0.539 | 2.544 | 0.091 |
| Dose × obstruction | 2 | 1.698 | 8.014 | 0.002 |
| Lack of fit | 3 | 0.041 | 0.193 | 0.900 |
| Residual | 36 | 0.212 | ... | ... |

The mathematical model fitted is shown below. The figures in brackets are the standard errors of the model parameter estimates:

$$\log(\text{conc.}) = \underset{(0.66)}{\underline{(-0.652 \times \log t)}} + K \qquad \text{where } t = \text{time in minutes}$$

where K is obtained from the following table:

(ii)

| Dose | 100 mg | | | 200 mg | | |
|---|---|---|---|---|---|---|
| Obstruction | Nil | Partial | Complete | Nil | Partial | Complete |
| K | 2.590 | 2.382 | 2.844 | 3.087 | 4.496 | 3.894 |
| (Standard error) | (0.312) | (0.367) | (0.392) | (0.325) | (0.429) | (0.551) |

Example time = 30 minutes, dose = 100 mg., obstruction = partial
Then log(conc) = (−0.652 − log(30)) + 2.382 = 0.164
Thus estimated blood conc. at 30 minutes on 100 mg = 1.178 mg/l.

### *Common Bile Duct*

The prediction model for common bile duct concentrations is detailed in Table III (i and ii). The agreement between the model predictions and the observed assay values are shown graphically in Figures 3 and 4. As for the venous blood data, only the prediction curve for unobstructed common bile ducts is reported for the 200 mg dose level.

Again, ciprofloxacin concentration fell exponentially with time, and concentrations were consistently higher with the 200 mg dose than with the 100 mg dose. On both doses the concentrations decreased significantly as the degree of obstruction increased.

### *Gall Bladder Bile*

In four patients no sample was obtained due to biliary sludge (3) or previous cholecystectomy (1). In 11 patients ciprofloxacin concentration was greater in gall bladder bile than in common bile duct bile and in 2 it was 18 times that found in the common duct. In 7 of the 11 patients a pre-operative oral cholecystogram was available showing a functioning gall bladder. The other 4 had pre-operative ultrasound only for diagnosis of gallstones.

FIGURE 1

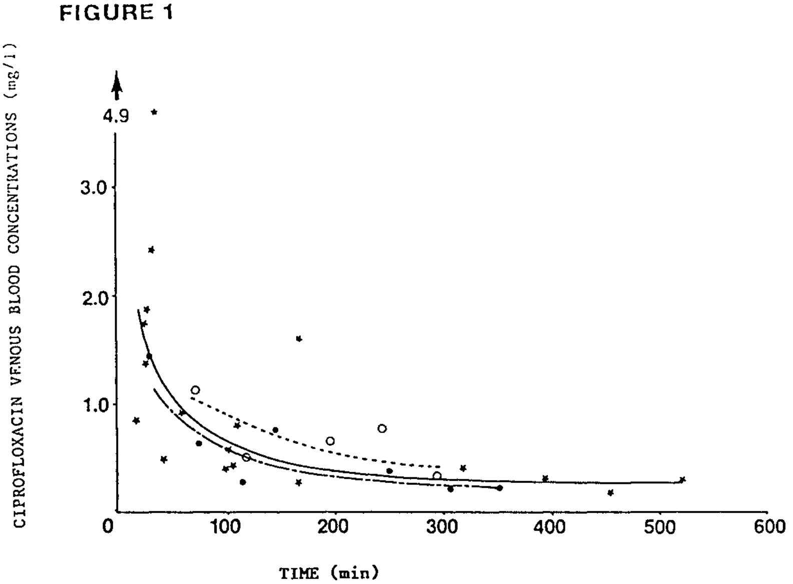

**Figure 1** Ciprofloxacin venous blood level (mg/l) following a 100mg iv bolus dose, administered at varying times up to 8 hours before induction of anaesthesia, in patients with unobstructed (*) partially obstructed (●) or completely obstructed (o) common bile ducts (CBDs). Comparison with predicted ciprofloxacin venous blood levels for unobstructed (————), partially obstructed ( ——— ) and completely obstructed (------) CBDs.

FIGURE 2

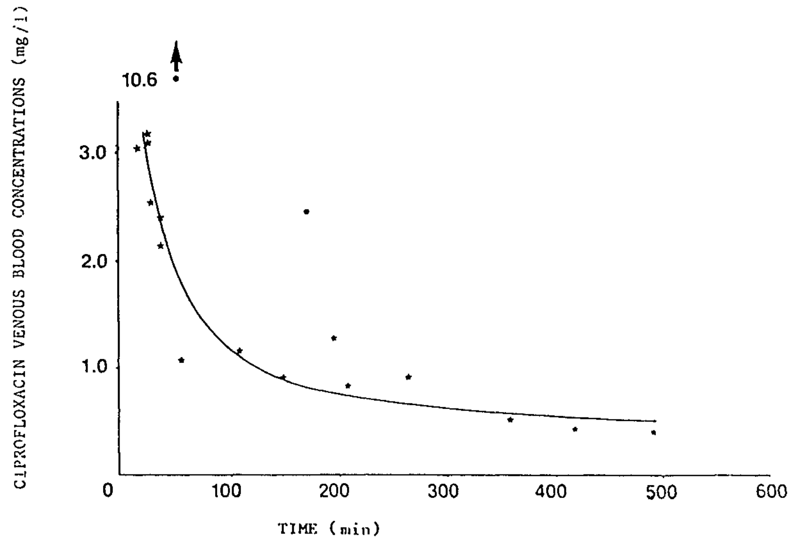

**Figure 2** Ciprofloxacin venous blood levels (mg/l) following a 200mg iv bolus dose, administered at varying times up to 8 hours before induction of anaesthesia, in patients with unobstructed (*), partially obstructed (●) or completely obstructed (o) CBDs. Comparison with predicted ciprofloxacin venous blood levels for unobstructed CBDs (————).

**Table 3** Analysis of common bile duct concentrations – derivation of predicted values

(i) *Analysis of variance table*

| *Source* | Degrees of Freedom | Mean Square | F-ratio | Probability |
|---|---|---|---|---|
| Time | 1 | 16.373 | 68.373 | 0.001 |
| Dose | 1 | 1.834 | 7.692 | 0.009 |
| Obstruction | 2 | 3.913 | 16.412 | 0.001 |
| Dose × obstruction | 2 | 0.647 | 2.714 | 0.078 |
| Lack of fit | 3 | 0.602 | 2.526 | 0.072 |
| Residual | 36 | 0.238 | ... | ... |

The mathematical model fitted is shown below. The figures in brackets are the standard errors of the model parameter estimates:

$$\log(\text{conc.}) = \underset{(0.075)}{\underline{(-0.489 \times \log t)}} + K \qquad \text{where } t = \text{time in minutes}$$

where K is obtained from the following table:

(ii)

| Dose | 100 mg | | | 200 mg | | |
|---|---|---|---|---|---|---|
| Obstruction | Nil | Partial | Complete | Nil | Partial | Complete |
| K<br>(Standard error) | 3.727<br>(0.357) | 3.335<br>(0.402) | 2.619<br>(0.436) | 4.154<br>(0.358) | 3.762<br>(0.418) | 3.046<br>(0.454) |

Example: time = 30 minutes, dose = 100 mg., obstruction = partial
Then: log(conc) = (−0.489 × log(30)) + 3.335 = 1.672
Thus: estimated blood conc. at 30 minutes on 100 mg = 5.323 mg/l.

### DISCUSSION

The high serum concentrations of ciprofloxacin fell rapidly during the first hour after infusion. Higher concentrations were maintained for longer with the 200 mg dose than with the 100 mg dose. This early fall in serum concentrations may reflect the rapid dispersal of the drug due to its exceptional tissue penetration[8]. The antibiotic rapidly occupies a large volume of distribution (2 1/kg) and after 4 hours decreasing serum concentrations are due solely to elimination of the drug. The kidneys are the major route of elimination of ciprofloxacin and its clearance is twice that of creatinine, indicating active tubular secretion[9].

Studies with other antibiotics have shown serum concentrations to be increased by duct obstruction[10]. A similar phenomenon was observed in this study but the magnitude was less suggesting that biliary excretion of ciprofloxacin is a minor contribution to the rapid fall in serum concentration when compared with diffusion into the tissues.

An alternative explanation is that ciprofloxacin excreted in the bile is mainly reabsorbed by the enterohepatic circulation, thereby restoring falling serum levels.[11].

FIGURE 3

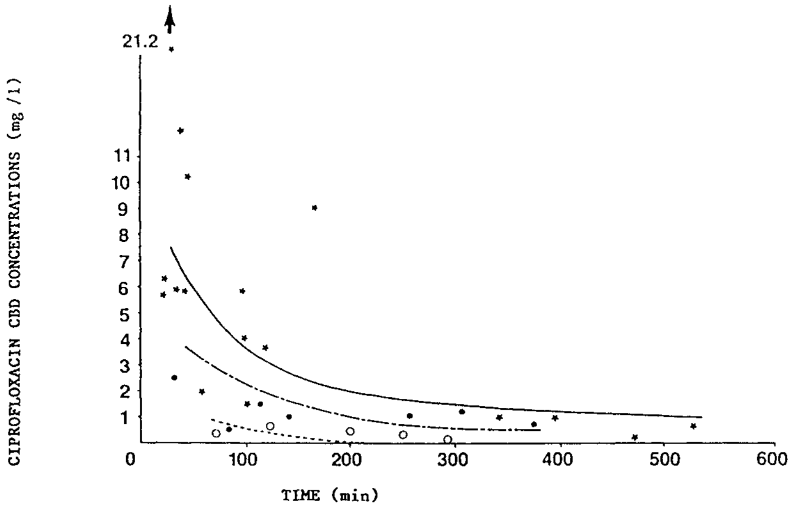

**Figure 3** Ciprofloxacin CBD concentrations (mg/l) following a 100mg iv bolus dose, administered at varying times up to 8 hours before anaesthetic induction, in patients with unobstructed (*) partially obstructed (•) or completely obstructed (o) CBDs. Comparison with predicted ciprofloxacin CBD levels for unobstructed (————), partially obstructed ( ——— ) and completely obstructed (------) CBDs.

FIGURE 4

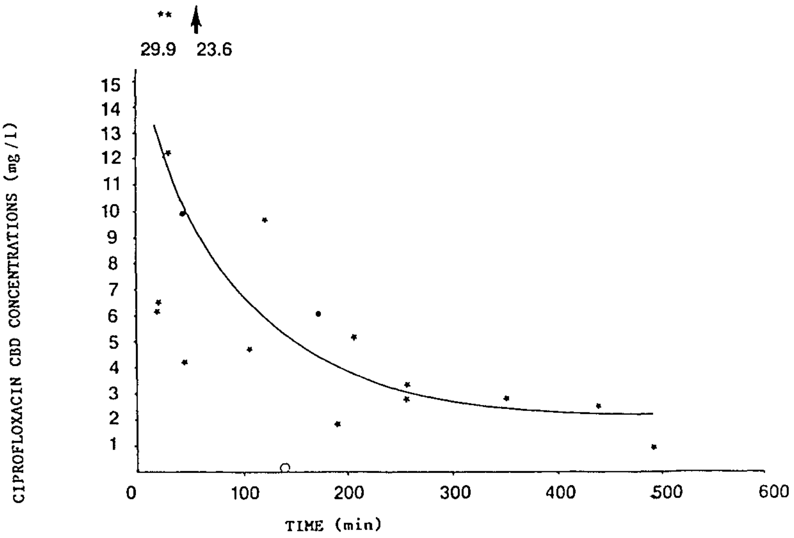

**Figure 4** Ciprofloxacin CBD concentrations (mg/l) following a 200mg iv bolus dose, administered at varying times up to 8 hours before anaesthetic induction, in patients with unobstructed (*), partially obstructed (•) or completely obstructed (o) CBDs. Comparison with predicted ciprofloxacin CBD levels for unobstructed (————) CBDs.

At both the doses studied, ciprofloxacin appeared in high concentration in common duct bile within 20 minutes of its infusion. The early decay of the exponential curves probably reflects the rapid diffusion of the drug into the tissues. However, useful concentrations were maintained for up to 8 hours particularly after a 200 mg dose. It would appear that one hour before anaesthetic induction would be the ideal time to give this antibiotic as an intravenous infusion. In both groups common duct bile concentration decreased as the degree of obstruction increased. A remarkable feature of this study was the finding of clinically significant concentrations of the antibiotic in the bile from ducts that had been completely obstructed for up to two months. Perhaps the antibiotic diffuses directly into the common bile duct from the rich arterial plexuses of the common duct wall.

This study was designed to assess the pharmocokinetics of biliary extretion of this antibiotic and not prophylactic potential, but the results have relevance to prophylaxis. *Escherichia coli, Klebsiella* and *Proteus* account for about 90% of the organisms cultured from bile and ciprofloxacin exhibits good activity against these organisms, the $MIC_{90}$ being less than 0.25 mg/l[12]. These organisms and *Staphylococcus aureus* from the skin of the patient and his attendants are responsible for more than 95% of wound infections following biliary surgery. A variety of other organisms may be found infrequently in the bile. Of these infrequent pathogens, *Streptococcus faecalis* and clostridia are found more frequently. These two organisms have an $MIC_{90}$ for ciprofloxacin of 2 mg/l.

*Streptococcus faecalis* has been cultured occasionally from an infected wound but we have never isolated clostridia from a wound infection following biliary surgery. Ciprofloxacin shows poor activity against *Bacteriodes fragilis* which has an $MIC_{90}$ of 8 mg/l but fortunately this is a rare pathogen in bile[1,3]. A concentration of 2 mg/l should therefore protect against all but the rare anaerobic pathogens. In common duct bile this level was achieved within 20 minutes at both doses, and in the majority of functioning gall bladders this level was greatly exceeded. With the 100 mg dose common duct concentrations fell below 2 mg/l after 3.5 hours, but with 200 mg remained above this level for 8.5 hours. Concentrations of 0.25 mg/l should protect against 90% of biliary pathogens, a level exceeded for over 8 hours in partial duct obstruction and for over 2 hours when complete obstruction was present. Although simple cholecystectomy should take less than an hour, complex biliary operations where the bile is inevitably infected may take 6 hours. Tissue concentration, although not measured in the study, has been shown to reach twice the serum concentration[8]. For prophylaxis in biliary tract surgery, serum and tissue concentrations may be as important as those in the bile[13]. The number of patients studied was too small for the mathematical models reported to be considered as sufficiently accurate for predicting ciprofloxacin concentrations in individual patients. A much larger series would be required to achieve that and the statistical analysis would have to be extended to take into account any important characteristics (e.g. age, sex, weight) which may influence rate of dispersal.

*Acknowledgements*

Ciprofloxacin levels were assayed by Dr. L. O. White PhD, Principal Scientific Officer in the laboratory of Dr. D. S. Reeves, MB, FRCPath, Consultant Medical Microbiologist at Southmead Hospital, Bristol.

### References

1. Keighley, M.R.B. (1977) Micro-organisms in the bile. *Annals of the Royal College of Surgeons,* **59**, 329–334
2. Strachan, C.J.L., Black, J., Powis, S.J.A., Waterworth, T.A., Wise, R., Wilkinson, A.R., Burdon, D.W., Severn, M., Mitra, B. and Norcott, H. (1977) Prophylactic use of cephazolin against wound sepsis after cholecystectomy. *British Medical Journal,* **i**, 1254–1256
3. Gallagher, P., Ostick, G., Jones, D., Schofield, P.F. and Tweedle, D.E.F. (1982) Intraoperative microscopy of bile – is it useful? *British Journal of Surgery,* **69**, 473–474
4. Papachristodoulou, A.J., Mackenzie, A., Norman, J. and Karran, S.J. (1978) Single dose cephazolin prophylaxis in biliary tract surgery. *Journal of the Royal College of Surgeons, Edinburgh,* **23**, 178–183
5. Ball, A.P. (1986) Overview of clinical experience with ciprofloxacin. *European Journal of Clinical Microbiology,* **5**, 214–219
6. Aitken, M.A. (1978) The analysis of unbalanced cross-classifications (with discussion). *Journal of the Royal Statistical Society, A,* **141**, 195–223
7. Baker, R.J. and Nelde, J.A. (1978) The GLIM System, Release 3. Oxford: Numerical Algorithms Group.
8. Crump, B., Wise, R. and Dent, J. (1983) The pharmacokinetics and tissue penetration of ciprofloxacin. *Antimicrobial Agents and Chemotherapy,* **24**, 784–786
9. Wingender, W., Graefe, K-H., Gau, W., Forster, D., Beerman, D. and Schacht, P. (1984) Pharmacokinetics of ciprofloxacin after oral and intravenous administration in healthy volunteers. *European Journal of Clinical Microbiology,* **3**, 355–359
10. Owen, A.W.M.C., Manson, J. McK., Yates, R.A., Adshead, V.M., Houghton, H.L. and Tweedle, D.E.F. (1983) The pharmacokinetics of cefotetan excretion in the unobstructed biliary tree. *Journal of Antimicrobial Chemotherapy,* **11** (Suppl. A) 217–221
11. Shah, P.M. (1985) Enterohepatic circulation of ciprofloxacin? Zeitschrift fur Antimikrobielle und Antineoplastische Chemotherapie, **3**, 85–86
12. Fass, R.J. (1983) In vitro activity of ciprofloxacin. Antimicrobial Agents and Chemotherapy, **24**, 568–574
13. Thomas, M. (1983) Antibiotics in bile. *Journal of Antimicrobial Chemotherapy,* **12**, 419–421

*(Accepted by S. Bengmark on January 1, 1989)*

### INVITED COMMENTARY

The quinolone group of antimicrobial agents will unquestionably play a major part in the treatment and prophylaxis of patient requiring hepatobiliary surgery. This paper examines the pharmacokinetics of 100 and 200 mg intravenous infusions of Ciprofloxacin in patients undergoing biliary surgery. The incidence of clinical sepsis was low. The concentrations of Ciprofloxacin achieved in serum, common bile duct and gall bladder bile were high and reasonably well sustained. As in all other publications in which the pharmacokinetics of antimicrobials in biliary disease have been examined, levels were inversely related to the degree of obstruction.

The unique feature of this communication relates to a mathematical model which has been used to predict serum and bile duct concentrations depending on the time of administration, the dose of antimicrobial and the degree of obstruction. Unfortunately, this last variable is subjective using the analysis designed for these predictive parameters. More helpful would have been a model which had related serum and bile levels to serum bilirubin concentration. Nevertheless, the model seems to have been successful in non-obstructive patients. It is a pity that the numbers of patients with bile duct obstruction were too small to allow numerical analysis of their data. It is, of course, patients with bile duct obstruction that present the highest risk from infection in biliary surgery and it is precisely these patients in whom a mathematical model should be tested.

The data presented by these authors is by no means unique. Serum and bile concentrations of Ciprofloxacin have been reported by other groups. More importantly, tissue concentrations, which seem to be very high with the quinolones, have also been clearly identified by other authors. It is a pity that these investigators have not taken the opportunity to assay levels of Ciprofloxacin in liver gall bladder wall and in the pancreas amongst patients requiring pancreatic resection. Despite these criticisms, the contribution is an important one and marks real progress in new agents which have considerable therapeutic potential for hepatobiliary infections.

M R B Keighley MS FRCS

